# Outcomes of Hospital Transfers for Pediatric Abdominal Pain and Appendicitis

**DOI:** 10.1001/jamanetworkopen.2018.3249

**Published:** 2018-10-12

**Authors:** Urbano L. França, Michael L. McManus

**Affiliations:** 1 Division of Critical Care, Department of Anesthesiology, Critical Care and Pain Medicine, Boston Children’s Hospital, Boston, Massachusetts; 2 Harvard Medical School, Boston, Massachusetts

## Abstract

**Importance:**

Hospital care for children is becoming more concentrated, with interhospital transfer occurring more frequently even for common conditions. Condition-specific analysis is required to determine the value, costs, and consequences of this trend.

**Objectives:**

To describe the capabilities of transferring and receiving hospitals and to determine how often children transferred after an initial diagnosis of abdominal pain or appendicitis require higher levels of care.

**Design, Setting, and Participants:**

Retrospective cohort analysis using the 2 most recent available inpatient and emergency department administrative data sets from all acute care hospitals in California from 2010 to 2011 and Florida, Massachusetts, and New York from 2013 to 2014. Data were analyzed between February and June 2018. All patients younger than 18 years with a primary diagnosis of abdominal pain or appendicitis who underwent an interhospital transfer and whose care could be matched through unique identifiers were included.

**Main Outcomes and Measures:**

Outcomes after hospital transfers, classified into encounters with major surgical procedures, imaging diagnostics, and no major procedures. Pediatric Hospital Capability Index of transferring and receiving hospitals.

**Results:**

There were 465 143 pediatric hospital encounters for abdominal pain and appendicitis, including 53 517 inpatient admissions and 15 275 transfers. Among them, 4469 could be matched to encounters in receiving hospitals. The median (interquartile range) age of this cohort was 10 (7-14) years, with 54.8% female (2449 patients), 40.9% male (1830 patients), and 4.3% unreported sex (190 patients). The increase in capability at the receiving hospital compared with the transferring hospital was large (median [interquartile range] change in Pediatric Hospital Capability Index score, 0.70 [0.54-0.82]), with 9.2% of hospitals (57) with very high capability (Pediatric Hospital Capability Index score >0.77) receiving 80.8% of the total transfers (3610). Diagnostic imaging was undertaken in the care of 710 transferred patients (15.9%) and invasive procedures were performed in 2421 patients (54.2%), including 2153 appendectomies. No imaging or surgery was required in the care of 1338 transfers (29.9%).

**Conclusions and Relevance:**

In this study, interfacility transfers of patients with appendicitis and abdominal pain were concentrated toward high-capability hospitals, and about 30% of patients were released without apparent intervention. These findings suggest an opportunity for improving care and decreasing cost through better interfacility coordination, such as standardized management protocols and telemedicine with high-capability hospitals. Further research is needed to identify similar opportunities among other common conditions.

## Key Points

**Question:**

How often do children transferred for management of abdominal pain and appendicitis require specialized pediatric services?

**Findings:**

This cohort study found that children with abdominal pain or suspected appendicitis were regularly transferred from hospitals with a wide range of capabilities to a small subset of highly capable centers. Although more than half (54.2%) required surgery, nearly one-third (29.9%) were discharged from the receiving hospital without intervention.

**Meaning:**

Improved coordination between high-capability and lower-capability hospitals may decrease the cost and increase the quality of care for children with abdominal pain and suspected appendicitis.

## Teaser

This cohort study examines the outcomes of children transferred to high-capability hospitals for treatment of abdominal pain and appendicitis.

## Introduction

Despite decreasing numbers of admissions, interfacility transfer is increasingly common among children requiring hospital care.^1,2^ This phenomenon can be viewed as part of a larger trend toward concentration of pediatric medical and surgical care within specialized centers.^3,4^ While regionalization of care into specialized centers promises improved experiences and outcomes for high-risk, complex, or uncommon conditions, the potential benefits are less obvious for routine conditions. Detailed study of regionalization outcomes is therefore needed.^5^

Changes in sites of care represent the summation of a variety of factors, including changes in the complexity of patients,^6^ changes in the abilities of hospitals,^7^ changes in specialty referral patterns,^8^ network constraints among insurers,^9,10^ and personal choices of families.^11^ For some conditions in some settings, these forces may work to drive improvements in cost, quality, and access to care.^12,13^ For other conditions, they may produce waste, inefficiencies, access delays, and diminished care quality.^14,15,16,17^ Uncertainty around these competing possibilities requires better understanding of the condition-specific risks, benefits, and costs of hospital transfer.^18^

While families and insurance purchasers may assume that local hospitals meet the routine needs of children, common conditions account for the largest increase in pediatric transfers and hospital care regionalization.^4^ Among these, treatment of appendicitis and abdominal pain has recently experienced one of the greatest shifts toward more specialized centers.^4^ To better understand the nature and value of this shift, we investigated the sources and outcome of interhospital transfer of children with abdominal pain and appendicitis within 4 US states composing approximately 25% of the US population.

## Methods

### Data Source

Hospital encounters in California from 2010 to 2011 and Florida, Massachusetts, and New York from 2013 to 2014 were identified within the Healthcare Cost and Utilization Project (HCUP) state inpatient^19^ and emergency department (ED)^20^ data sets and the Massachusetts Center for Health and Information and Analysis inpatient, observation admission, and ED acute hospital case mix data sets.^21^ The HCUP databases are aggregated and maintained by the Agency for Healthcare Research and Quality in partnership with state and private organizations. They include encounter-level, demographic, and clinical information concerning all ED visits and hospital admissions within a state. The Center for Health and Information and Analysis case mix data sets contain similar information regarding hospital care within the Commonwealth of Massachusetts. All data sets are available to facilitate public health, policy, and biomedical research. Our proposed use of this data was reviewed and approved by the Center for Health and Information and Analysis Data Release Committee. Data use approval and a waiver of informed consent were also obtained from the Boston Children’s Hospital Committee on Clinical Investigation. The study followed the Strengthening the Reporting of Observational Studies in Epidemiology (STROBE) reporting guideline.

### Population and Transfer Matching

The target population included pediatric patients (aged <18 years) who underwent hospital transfer after an initial encounter primary diagnosis of appendicitis or abdominal pain (Clinical Classifications Software codes^22^ 142 and 251). Patients were identified by diagnosis and transfer disposition within referral hospitals and then matched to receiving hospital encounters using unique synthetic identifiers according to HCUP recommendations.^23^ The availability of unique identifiers varies widely by state and tends to increase with age. Patients without unique identifiers and those who could not be matched to a specific subsequent encounter were not included.

### Outcome of Transfers and Cost

The outcome of matched transfers was characterized according to activity within receiving hospitals, including admission, discharge, diagnostic or therapeutic procedures, and discharge diagnosis. Procedures were identified through review of all *Current Procedural Terminology* and *International Classification of Diseases, Ninth Revision,* codes included within each encounter. Where available, charges among receiving hospitals were totaled, averaged, and adjusted for inflation to 2017 US dollars using information from the Bureau of Labor Statistics.^24^ Costs were also estimated using HCUP’s cost-to-charge ratio files.^25^ To understand the role of age in the outcome of transfers, patients were grouped into 4 age groups: 0 to 4 years, 5 to 9 years, 10 to 14 years, and 15 to 17 years.

### Comparison of Hospitals

To compare the nature of referring and receiving hospitals, we calculated the pediatric Hospital Capability Index (pHCI) score for each hospital during the 2-year period of study. As described and applied elsewhere, the HCI score is between 0 and 1, quantifies a hospital’s average completion of care across all Clinical Classifications Software conditions, and may be stratified by age or other variables of interest.^26^ Hospitals with HCI scores closer to 1 admit and care for patients with a broad range of conditions and transfer infrequently. Hospitals with HCI scores closer to 0 care for fewer conditions and transfer often. Pediatric HCI scores were calculated based on hospital overall experience with patients younger than 18 years. Full details of these calculations are included in the eAppendix in the Supplement.

### Statistical Analysis

We report descriptive statistics for total charges of patients, insurance status, and differences in pHCI score. Total costs of the different groups and ages between matched transfers and total transfers with unique identifiers were compared using the Mann-Whitney *U* test. The frequencies of types of discharges among age groups were compared using Monte Carlo resampling. The comparison between transfers to hospitals with high and low pHCI scores and patient sex and race between matched transfers and total transfers with unique identifiers was performed using the χ^2^ test. Bootstrapping was used to estimate significance of pHCI score comparison between receiving and transferring hospitals. All analyses used Python 3.6, an open-source programming language, and the Jupyter environment. All statistical tests were 2-tailed, and significance was set at *P* < .05.

## Results

**Figure 1.**
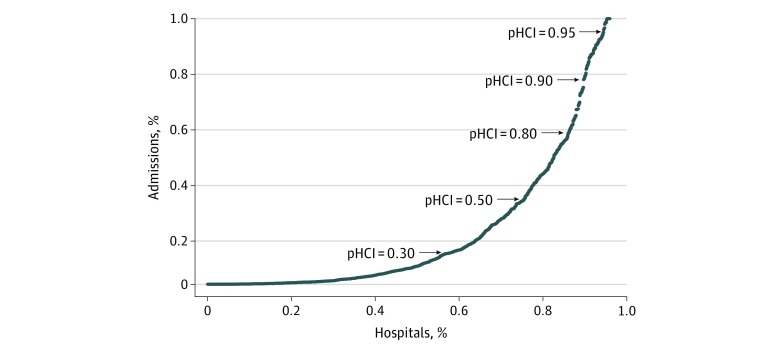
Cumulative Percentage of Pediatric Hospital Admissions for Abdominal Pain and Appendicitis The percentages are ordered according to pediatric Hospital Capability Index (pHCI), which varies between 0 (low pediatric capability on average) and 1 (high pediatric capability on average). Discontinuities in the curve reflect the fact that some hospitals admit a significant proportion of pediatric patients.

Within the 4 states over the 2 years of study, there were 465 143 pediatric hospital encounters for abdominal pain and appendicitis, resulting in 53 517 admissions and 15 275 transfers. Among the transferred patients, 2137 (14.4%) were aged 0 to 4 years, 4887 (32.9%) were aged 5 to 9 years, 5269 (35.5%) were aged 10 to 14 years, and 2540 (17.4%) were aged 15 to 17 years. In total, 746 hospitals encountered children with abdominal pain and appendicitis, although many reported no admissions. Overall, approximately 65% of all admissions were to the roughly 20% of hospitals with pHCI scores of 0.52 or greater. However, there was high variability in the number of admissions per hospital and a significant number of admissions concentrated in the very high–capability centers (Figure 1).

Unique identifiers were available in 49.3%, 63.6%, 48.1%, and 89.6% of encounters in California, Florida, Massachusetts, and New York, respectively. Of the 280 189 encounters where unique identifiers were present, 8703 (3.1%) involved patient transfer. Among those transferred, 4469 (51.3%) patients could be definitively matched to resulting encounters within receiving hospitals. The median (interquartile range [IQR]) age of the patients in this cohort was 10 (7-14) years. Most of the patients were white (70.6% [3155 patients]); 54.8% were female (2449 patients), 40.9% were male (1830 patients), and 4.3% (190 patients) had no sex reported. Demographic information per state is included in Table 1. These demographic characteristics (age, sex, and race) of matched transfers did not differ significantly from the larger population of all transfers with identifiers (*P* > .10 for all comparisons).

**Table 1.** Characteristics of Children Transferred and Encounters for Abdominal Pain or Appendicitis

Patient and Stay Characteristics	No. (%)				
	California (2010-2011)	Florida (2013-2014)	Massachusetts (2013-2014)	New York (2013-2014)	All States
Transfers from emergency department, No.	1241	1814	268	1146	4469
Demographic characteristics					
Age, median (IQR)	9 (7-12)	11 (8-15)	11 (8-14)	10 (6-13)	10 (7-14)
Sex					
Male	440 (35.5)	750 (41.3)	108 (40.3)	532 (46.4)	1830 (40.9)
Female	611 (49.2)	1064 (58.7)	160 (59.7)	614 (53.6)	2449 (54.8)
Unknown	190 (15.3)	0	0	0	190 (4.3)
Race					
White	767 (61.8)	1482 (81.7)	146 (54.5)	760 (66.3)	3155 (70.6)
Black or African American	53 (4.3)	205 (11.3)	30 (11.2)	132 (11.5)	420 (9.4)
Other or unknown	421 (35.9)	127 (7.0)	92 (34.3)	254 (22.1)	894 (20.0)
Length of stay, d					
<1	301 (24.3)	444 (24.5)	123 (45.9)	458 (40.0)	1326 (29.7)
1	330 (26.6)	630 (34.7)	97 (36.2)	332 (29.0)	1389 (31.1)
≥2	610 (49.1)	740 (40.8)	48 (17.9)	356 (31.0)	1754 (39.2)
Inflation-adjusted cost, median (IQR), $					
Emergency department	NA^a^	2406.85 (675.90-5995.72)	908.23 (549.59-1423.06)	994.48 (557.73-2327.08)	1256.31 (591.54-4362.70)
Admissions	7517.79 (4353.33-11 389.47)	6676.23 (4146.84-9664.09)	5912.28 (2617.76-9189.42)	6535.62 (4306.01-9759.40)	6820.00 (4150.61-10 263.02)

Abbreviations: IQR, interquartile range; NA, not available.

^a^ Charges are not available in the Healthcare Cost and Utilization Project emergency department data set in California.

Within the study population of matched transfers, 1626 (36.4%) were discharged directly from the receiving ED and 2843 (63.6%) were admitted to the receiving hospital. Among admitted patients, 1160 (25.9%) were discharged within 24 hours and 1683 (37.7%) required stays of 2 days or more (for additional information, see Table 1). Diagnostic imaging, such as radiography, computed tomography, or ultrasonography, was undertaken in the care of 710 transferred patients (15.9%). Invasive procedures were performed in 2421 patients (54.2%), including 2153 appendectomies. No imaging or surgery was required in the care of 591 transferred patients (13.2%) who were released directly from the receiving ED and 747 (16.7%) who were admitted to the hospital but discharged without any procedures (1338 patients [29.9%], total) (eFigure in the Supplement). Overall diagnostic and therapeutic interventions required among transferred patients within each of the hospital settings are summarized in Table 2.

**Table 2.** Top Clinical Classifications Software Procedure Codes for Imaging Diagnostics and Surgical Procedures

Procedure Types	No. (%)
Imaging only, No.	710
Diagnostic ultrasonography	505 (71.1)
Radiography of abdomen	111 (15.6)
Computed tomographic scan of abdomen	40 (5.6)
Endoscopy and biopsy	18 (2.5)
Other procedures^a^	36 (5.1)
Procedures, No.	2421
Appendectomy	2153 (88.9)
Endoscopy and biopsy	44 (1.8)
Abdominal paracentesis	36 (1.5)
Drainage of appendiceal abscess	24 (1.0)
Laparoscopy	14 (0.6)
Cholecystectomy	13 (0.6)
Other procedures^a^	137 (5.6)

^a^ The category other procedures includes those with fewer than 11 counts per procedure.

**Figure 2.**
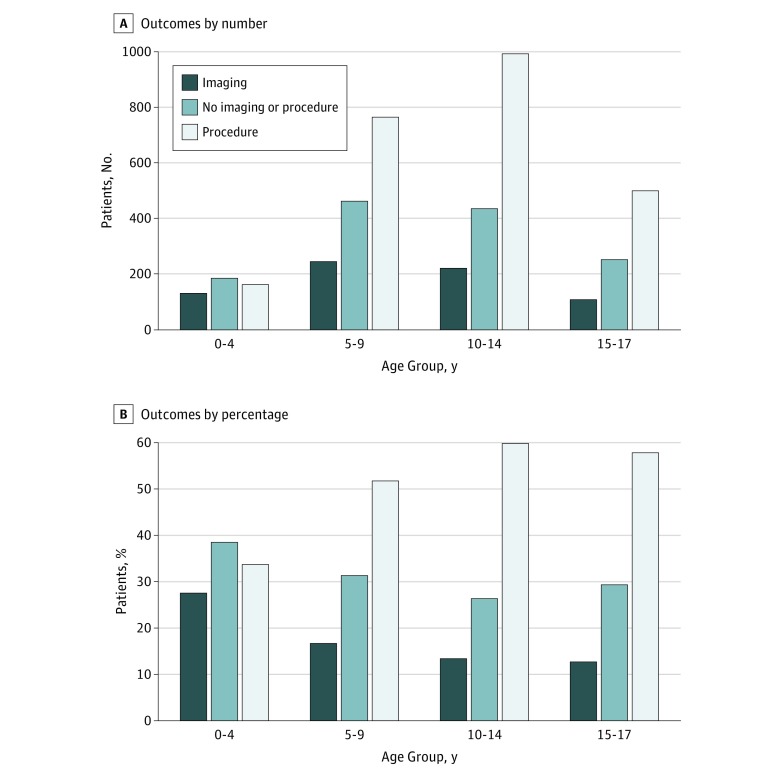
Outcomes by Age Group Number (A) and percentage (B) of outcomes at the receiving hospital for patients in 4 age groups.

As presented in Figure 2, ultimate outcomes differed significantly (*P* < .001) by age, with the youngest children least likely to require surgery (33.8% for age 0-4 years vs 51.9% for age 5-9 years, 60.2% for age 10-14 years, and 58.0% for age 15-17 years) and most likely to be discharged without intervention (38.6% for age 0-4 years vs 31.4% for age 5-9 years, 26.4% for age 10-14 years, and 29.4% for age 15-17 years). The youngest children also constituted the smallest fraction of total transfers, with school-aged children representing more than two-thirds of the total (14.4% for age 0-4 years, 32.9% for age 5-9 years, 35.5% for age 10-14 years, and 17.1% for age 15-17 years).

**Figure 3.**
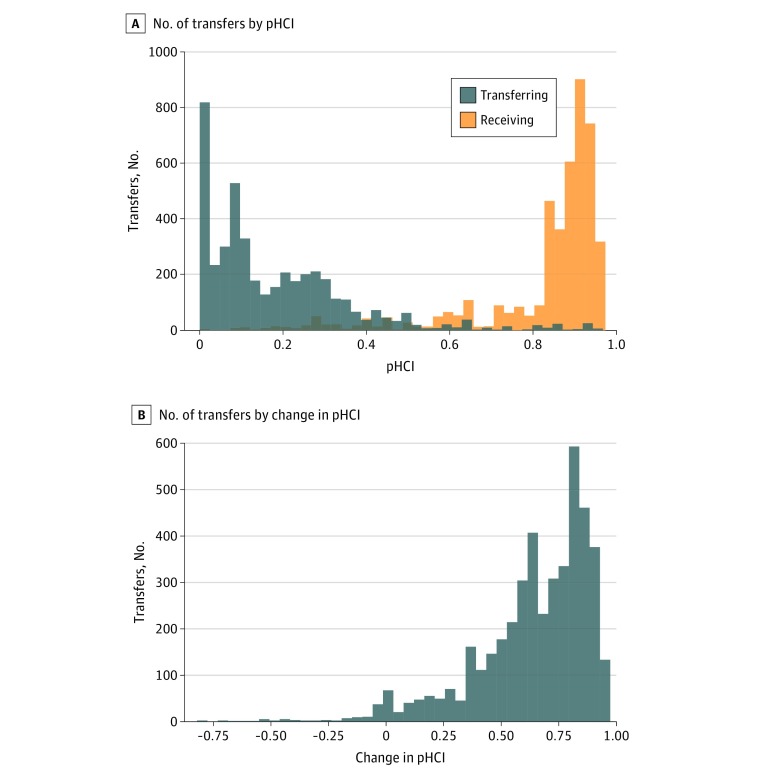
Distribution of Transfers by Pediatric Hospital Capability Index (pHCI) and Change in pHCI A, Distributions of transferring hospitals and receiving hospitals according to pHCI. B, Distribution of pHCI score change between the receiving and transferring hospitals. The pHCI scores range between 0 (low average capability) and 1 (high average capability).

Matched patients visited a total of 620 hospitals, with 548 transferring hospitals and 219 receiving hospitals (including 147 hospitals that both transferred and received patients). Some hospitals received few transfers, yet 80.8% of all transfers (3610) were directed to a small subset of hospitals (57 [9.2%]) with very high capability (pHCI score >0.77) (Figure 3). The typical increase in capability at the receiving hospital was large (median [IQR] change in pHCI score, 0.70 [0.54-0.82]) (*P* = .02), and only a small fraction of transfers (96 [2.1%]) were to hospitals with similar or lower pHCI scores. Close inspection of these suggested that they reflect within-system transfers, including 25 between a single pair of hospitals. There was no suggestion of insurance bias, as the distribution of insurance status was similar between patients transferred to hospitals with higher pHCI scores and patients transferred to hospitals with lower capability (60% vs 61% for Medicaid and 37% vs 36% for private insurance or self-pay; *P* = .99).

Primary discharge diagnoses at transferring and receiving hospitals agreed in 2438 transfers (54.6%) and disagreed in 2031 (45.4%). In 1764 transfers, there was concordance in the diagnosis of appendicitis. Among transfers with discordant diagnoses, 516 (25.4%) were ultimately discharged with a diagnosis of appendicitis after referral for abdominal pain, and 161 (7.9%) were discharged with a diagnosis of abdominal pain after referral for appendicitis. Additional discordances involved a variety of specific and nonspecific conditions, including other gastrointestinal disorders, gastroenteritis, lymphadenitis, urinary tract infection, intestinal obstruction, nausea/vomiting, and ovarian cyst.

Charges were available for more than 99.8% of the encounters in all states but California, where charges were unavailable for ED visits and 20.9% of admissions (207). Across all states, the median (IQR) cost for transferred patients sent home from the ED without imaging or surgical procedures was $482.30 ($343.41-$1031.23), and the median (IQR) cost for similar admitted patients was $2526.29 ($1493.34-$4678.48). Patients discharged after diagnostic imaging procedures had total median (IQR) costs of $1036.63 ($725.75-$1680.35) when discharged from the ED and $4948.21 ($2568.09-$7928.45) when discharged from inpatient settings. Patients who underwent surgical and other major procedures presented significantly higher costs both at the ED and inpatient settings (median [IQR], $5933.76 [$4650.07-$6929.12] and $8112.08 [$5846.27-$11 903.50], respectively; *P* < .001 for both ED and inpatient settings) (Table 1).

## Discussion

Interhospital transfer of children is increasing across the United States, and abdominal pain is among the conditions showing the greatest increase in transfer frequency. Our observations suggest that this is unrelated to changes in patient or need complexity because (1) nearly one-third of transferred patients are ultimately discharged from receiving hospitals without diagnostic or therapeutic intervention, (2) most transferred patients require either no admission or stays of less than 24 hours, (3) relatively few transfers are for the youngest children, and (4) most final discharge diagnoses from receiving hospitals suggest uncomplicated conditions. We additionally observed that transfers originated from a large number of hospitals with a wide range of capabilities but generally terminated within a very small number of highly capable centers. While two-thirds of patients received diagnostic imaging that may have been unavailable at their transferring hospitals, only about half of all transferred patients required surgical intervention.

Our findings are consistent with observations from several vantages that many hospital transfers ultimately prove unnecessary. Medford-Davis et al^27^ analyzed more than 48 000 ED-to-ED transfers of injured adults and also observed that more than one-third were released from receiving institutions without intervention. Li et al^28^ similarly found that approximately 42% of all interfacility transfers recorded within the Pediatric Health Information System database over a 12-month period were discharged directly from the ED or admitted for less than 24 hours. Bertazzoni and colleagues,^29^ studying a Roman hospital system serving 1.5 million people, looked at overall referral patterns and considered half of all transfers to be unjustified and dangerously contributing to hospital crowding. Even so, we observed that nearly 70% of all children transferred for abdominal pain or appendicitis required imaging or surgery at the receiving center and diagnostic concordance between referring and receiving institutions was high. In addition, transferred younger children, even when discharged without surgery or imaging, likely benefitted from the specialized physical assessments available in referral centers. Because most children with abdominal pain are still treated and released from community EDs, a false-negative transfer rate of approximately 30% may simply be the trade-off arising from regionalization of care.

The American College of Surgeons developed the Children’s Surgery Verification program to ensure that all infants and children requiring surgery “receive care in an environment with prospectively defined optimal resources matched to his/her needs.”^30^ Our findings suggest that an increasing number of children with abdominal pain or appendicitis are being admitted to higher-capability hospitals and that most transfers are to a subset of hospitals with very high capability. This is consistent with prior reports of a shift toward pediatric hospitals for certain procedures.^31^ However, our observations that approximately half of transferred children do not require surgery and that 29.9% of all transferred patients are ultimately released without any diagnostic or therapeutic intervention suggest an opportunity for improving care through interfacility cooperation. For example, when imaging capabilities are available in the community but specialized interpretation is not, telemedicine may reduce the risks, burdens, and costs of transfer.^32^ Transfers could also be reduced through better care coordination among children with special needs and standardized management protocols backed by access to specialty consultation.^33^ Finally, if appendicitis is suspected but travel distances are long, alternative care practices such as nonoperative management^34^ or treatment delay^35^ may offer options for managing risk without emergency transfer.

Although young children may be best served by the specialized services of high-capability children’s hospitals, the quality and safety requirements when caring for older children are less clear.^36,37^ Historically, most older children have been cared for in adult facilities,^37^ but we observed that most transfers for abdominal pain and appendicitis are now of older children and teenagers. While some parents bringing children to local hospitals may desire transfer to a specialized facility whenever surgery is contemplated, others may be surprised and disappointed by the need for transfer because evaluation and treatment of appendicitis has been among the least regionalized pediatric services.^4,31^ With some insurance products, transfer may also prove financially burdensome if it leads to out-of-network care.^10,38^

### Limitations

Our work carries all of the limitations of retrospective research involving large administrative data sets.^39^ In addition, these particular investigations are limited by the availability of reliable unique patient identifiers. Among HCUP state-specific data sets, unique identifiers are unavailable in most states and, when present, are inconsistently available among children. In addition, because the coding of discharge disposition and admission source is not always consistent with the timing of events, it can sometimes be difficult to definitively match 2 records as parts of a transfer.^23^ Here, we elected to follow a conservative path, relying on a large number of records across several states but including only those records that could be definitively matched. As a result, we have high confidence in the sources, destinations, and outcomes of the transfers reported, while acknowledging that the true number of transfers is much higher. Based on transfer source and disposition fields alone, we estimate true transfer frequencies to be as much as 4 times higher than those that could be matched. However, we believe that the study population reflects the total population because there was no indication of systematic bias and, within each state and age range, the matched and unmatched populations share similar distributions of sex, race, age, and insurance status. Our cost analysis was limited by the disproportionate availability of hospital charge information across states. We therefore elected to provide a state-specific cost analysis in Table 1, but make no definitive cost claims.

## Conclusions

Although interhospital transfer rates are increasing, our analysis of administrative data sets from 4 US states suggests that transfer of children with abdominal pain and appendicitis is largely unrelated to the complexity of need or intensity of services. Despite this, approximately 80% of all transfers are to very high–capability hospitals where approximately one-half require routine surgery and one-third are released without apparent intervention. The latter may represent an opportunity for decreasing cost and improving quality of care through better coordination among hospitals. Additional opportunities may also be revealed by similar analysis of other conditions.

## Notes

Supplement. **eAppendix.** Supplementary Information **eReferences** **eFigure.** Outcomes of Transferred Patients
