# Supplementary material for: Outcomes of Hospital Transfers for Pediatric Abdominal Pain and Appendicitis
Source: JAMA Netw Open. 2018 Oct 12;1(6):e183249. doi: 10.1001/jamanetworkopen.2018.3249 (PMC6324598; doi:10.1001/jamanetworkopen.2018.3249)
Supplement: Supplement. — eAppendix. Supplementary Information eReferences eFigure. Outcomes of Transferred Patients [file jamanetwopen-1-e183249-s001.pdf]

Supplementary Online Content

França UL, McManus ML. Outcomes of Hospital Transfers for Pediatric Abdominal Pain and Appendicitis. *JAMA Netw Open.* 2018;1(6):e183249. doi:10.1001/jamanetworkopen.2018.3249

eAppendix. Supplementary Information

eReferences

eFigure. Outcomes of Transferred Patients

This supplementary material has been provided by the authors to give readers additional information about their work.

© 2018 França UL et al. *JAMA Network Open.*

eAppendix. Supplementary Information

Hospital Capability and Regionalization Indices

We provide a brief overview of the pediatric Hospital Capability Indices (pHCI) used in the manuscript. A more complete discussion of the theoretical underpinnings and uses can be found at França & McManus (2017).[1] The index is based upon the *probability of care completion (P),* defined as the ratio of the number admissions to the sum of admissions *(A) and transfers (T) at a given* hospital:

$$\mathcal{P}(\mathrm{h}, \mathrm{CCS}, \mathrm{time}, \mathrm{age}) = \frac{A(\mathrm{h}, \mathrm{CCS}, \mathrm{time}, \mathrm{age})}{A(\mathrm{h}, \mathrm{CCS}, \mathrm{time}, \mathrm{age}) + T(\mathrm{h}, \mathrm{CCS}, \mathrm{time}, \mathrm{age})}$$

The probability of care completion is defined by hospital (*h*), period of time, and CCS code, which give the *minimal* set of variables, but additional ones, such as age, can be used to stratify the population. Because we are interested in quantifying the hospital capability for pediatric patients (<18 years) during the years 2013-2014, the equation above can be rewritten as

$$\mathcal{P}(\mathrm{h}, \mathrm{CCS}, 2013 \ \& \ 2014, \mathrm{age} < 18) = \frac{A(\mathrm{h}, \mathrm{CCS}, 2013 \ \& \ 2014, \mathrm{age} < 18)}{A(\mathrm{h}, \mathrm{CCS}, 2013 \ \& \ 2014, \mathrm{age} < 18) + T(\mathrm{h}, \mathrm{CCS}, 2013 \ \& \ 2014, \mathrm{age} < 18)}$$

With this probability we can define the pediatric Hospital Capability Index (pHCI), which quantifies the breadth of capability of a hospital to treat *all* medical conditions requiring at least 20 admissions in an acute care hospital during the period of interest. Following the discussion in Ref. 1 (see also the eAppendix of Ref. 2), the pHCI of a hospital is defined as:

$$\mathrm{pHCI}(\mathrm{h}, 2013 \ \& \ 2014, \mathrm{age} < 18) = \frac{\sum_{\mathrm{CCS}} \mathcal{P}(\mathrm{h}, \mathrm{CCS}, 2013 \ \& \ 2014, \mathrm{age} < 18)}{\mathrm{Number\ of\ CCS\ codes}(2013 \ \& \ 2014, \mathrm{age} < 18)}$$

eReferences

1. França UL, McManus ML. Transfer Frequency as a Measure of Hospital Capability and Regionalization. *Health Services Research* 2017;52(6):2237–2255. doi:10.1111/1475-6773.12583.

2. França UL, McManus ML. Availability of Definitive Hospital Care for Children. *JAMA Pediatr*. 2017;171(9):e171096. doi:10.1001/jamapediatrics.2017.1096.

© 2018 França UL et al. *JAMA Network Open.*

Outcomes of transferred patients

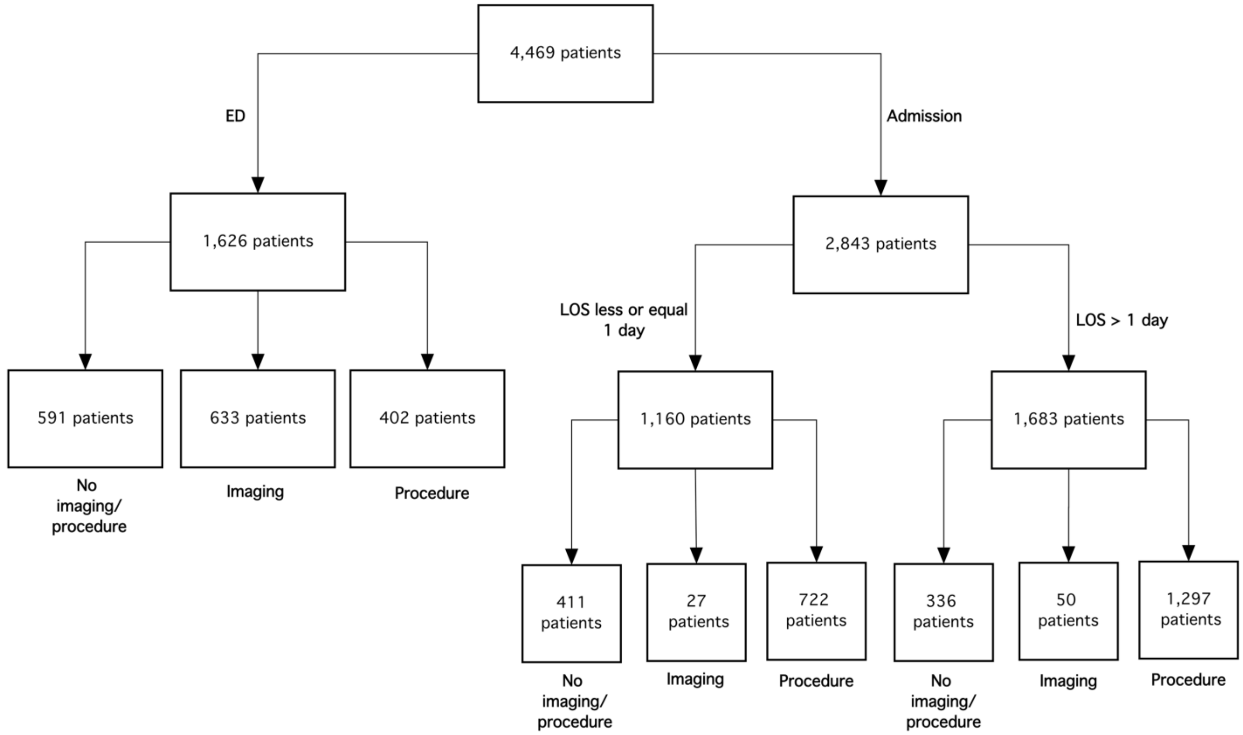

eFigure: Outcomes at the receiving hospitals for the patients in the study cohort based on the discharge hospital setting and outcome of hospital encounter. ED: Emergency department; LOS: Length of Stay.

© 2018 França UL et al. *JAMA Network Open.*
